# Obesity and Inflammation: Epidemiology, Risk Factors, and Markers of Inflammation

**DOI:** 10.1155/2013/678159

**Published:** 2013-04-17

**Authors:** Heriberto Rodríguez-Hernández, Luis E. Simental-Mendía, Gabriela Rodríguez-Ramírez, Miguel A. Reyes-Romero

**Affiliations:** ^1^Biomedical Research Unit of the Mexican Social Security Institute at Durango, Predio Canoas 100, Los Angeles, 34067 Durango, DGO, Mexico; ^2^Faculty of Medicine and Nutrition, Juárez University of Durango State, Av. Universidad and Fanny Anitúa s/n, Zona Centro, 34000 Durango, DGO, Mexico

## Abstract

Obesity is a public health problem that has reached epidemic proportions with an increasing worldwide prevalence. The global emergence of obesity increases the risk of developing chronic metabolic disorders. Thus, it is an economic issue that increased the costs of the comorbidities associated. Moreover, in recent years, it has been demonstrated that obesity is associated with chronic systemic inflammation, this status is conditioned by the innate immune system activation in adipose tissue that promotes an increase in the production and release of pro-inflammatory cytokines that contribute to the triggering of the systemic acute-phase response which is characterized by elevation of acute-phase protein levels. On this regard, low-grade chronic inflammation is a characteristic of various chronic diseases such as metabolic syndrome, cardiovascular disease, diabetes, hypertension, non-alcoholic fatty liver disease, and some cancers, among others, which are also characterized by obesity condition. Thus, a growing body of evidence supports the important role that is played by the inflammatory response in obesity condition and the pathogenesis of chronic diseases related.

## 1. Epidemiology and Obesity

Obesity is actually an epidemic problem in the world; it has become truly a global problem affecting countries rich and poor. An estimated 500 million adults worldwide are obese and 1.5 billion are overweight or obese [1]. Particularly the prevalence of obesity or combined overweight and obesity has increased in Brazil, Canada, Mexico, and United States [2]. Much of the information about obesity among adults rest in the use of body mass index (BMI) to define obesity, which will be defined as a BMI 30 kg/m^2^ or greater unless otherwise stated [3]. An examination of national data through 1991 confirmed that significant increases in the United States population had takes place both in adults and children and adolescents [4, 5]. The most recent data from 2005-2006 show that 33.3% of men and 35.3% of women were obese [6]. In Canada, the prevalence of obesity based on measured height and weight has almost doubled in the last two decades and now affects 23% of the adult population [7].

Obesity is a consequence of many risk factors, as increased energy consumption and reduced physical exercise. Many studies also implicate chronic low grade inflammation in the interplay between obesity and metabolic complications, as many chronic degenerative disorders, including atherosclerosis, and are also commonly associated with hypertension, which itself has also been linked recently to inflammation [8, 9]. Obesity and inflammation have been associated with type 2 diabetes, cardiovascular disease, hypertension, stroke, and gallbladder disease, some forms of cancer, osteoarthritis, and psychosocial problems [10]. In obesity subjects, this problem is commonly associated with other metabolic disorders as hyperglycemia and hypertriglyceridemia, which are well-known risk factors for developing chronic liver disease, as nonalcoholic fatty liver disease (NAFLD) [11, 12]. The prevalence of NAFLD reaches 14% to 21%, but is it as high as 90%–95% in obese persons and up to 70% in diabetic patients [13]. Liver inflammation can be induced by the metabolically active intraabdominal fat, and that the high BMI and large waist circumference are significantly associated with the elevation of aspartate aminotransferase (AST) and alanine aminotransferase (ALT) levels [14, 15]. Patients with obesity can have elevation of AST and ALT levels, and the reduction of body weight can be achieved with combining diet and physical activity strategies, and reduced levels of aminotransferase [16]. NAFLD and cardiovascular disease have common metabolic risk factors and have 3.7% on mortality; individuals with NAFLD were older, predominantly males, and more likely to be Hispanic. They also had a higher prevalence of all components of metabolic syndrome and cardiovascular disease; however, patients with NAFLD disease did not increase cardiovascular mortality in over 14 years [17]. 

For example, within the context of chronic HCV and HBV infection, the presence of cirrhosis is the most important risk factor in the development of hepatocellular carcinoma [18]. There are some nonmodifiable risk factors including older age, male gender, and family history, and several modifiable risk factors in hepatocelular carcinoma, of which the most important are alcohol and tobacco [19]. However, identifying additional modifiable risk factors, including diet, is important, including coffee and tea, fructose, iron, red and with meats, types of fat, selenium, and vitamins D and E [20].

Diet and life style play a crucial role in the development of some cancers. Actually in Mexico and others countries, more than one-third of cancer deaths can be avoided through dietary modification. Different mechanisms, including antioxidant, anti-inflammatory, and antiestrogenic processes, have been proposed to explain the protective nature of certain dietary components [21].

## 2. Obesity and Chronic Inflammation 

Inflammation is a physiological response necessary to restore homeostasis altered by diverse stimuli; however, inflammation state chronically established or an excessive response can involve deleterious effects. In overweight and obesity, there exists low-grade chronic inflammation; recent studies have unveiled some of the intracellular pathways of inflammation associated with these conditions; studies in mice and humans evidence that consumption of nutrients may acutely evoke inflammatory responses; so, it is thought that the starting signal of inflammation is overfeeding and the pathway origins in tissues involved in metabolism, that is, adipose tissue, liver, and muscle, which in response of this stimulus triggers the inflammatory response [22, 23]. Compared with lean control, in obese men and women, tissue and liver tissues display an increased activation of kinases such as c-jun N-terminal kinase and the inhibitor of k kinase, which are able to induce the expression of inflammatory cytokines [24, 25]. These kinases regulate downstream transcriptional programs through the transcription factors activator protein-1, nuclear factor *κ*B, and interferon regulatory factor, inducing upregulation of inflammatory mediator gene expression. The increase in cytokines exacerbates receptor activation by establishing a positive feedback loop of inflammation and the inhibitory signaling of metabolic pathways [26].

Likewise, inflammasome and the Toll-like receptors (TLRs) of the innate immune system are activated as well [27, 28]. Now, strong evidences indicate a prominent role of the inflammasome signaling in the development of a chronic proinflammatory state that impairs insulin sensitivity [24].

## 3. The Inflammasome

Inflammasome is a macromolecular innate immune cell sensor that initiates the inflammatory response. Recognition of diverse noxious signals by the inflammasome results in activation of caspase-1, which subsequently induces secretion of potent proinflammatory cytokines, particularly interleukin-1*β* (IL-1*β*). In this way, inflammasome-mediated processes are important in regulating metabolic processes [24, 29].

The inflammasome is a heptamer formed by monomers containing Nod-like receptors (NLRs), the adaptor protein ASC (apoptosis-associated speck-like protein containing a caspase-recruitment domain), and the enzyme caspase-1. NLRs are characterized by a structure composed of a central domain that mediates nucleotide-binding and oligomerization (NOD or NBS domain), a C-terminal leucine-rich domain (LRR), and a variable N-terminal region required for protein-protein interactions. When assembled as inflammasome, NLR activates caspase-1, which converts pro-IL-1*β* into active IL-1*β* [30, 31].

In the human being, the NLR family consists of 22 members, classified in 4 subfamilies, NLRA, NLRB, NLRC, and NLRP, on basis of their N-terminal domain configuration. They interact with the inflammasome-associated proteins ASC and caspase-1 [32]. 

A member of the NLRP, named NLRP3, has been linked to metabolic stress, insulin resistance, and type 2 diabetes. NLRP3 inflammasome activation in obesity promotes macrophage-mediated T cell activation in adipose tissue and impairs insulin sensitivity creating a chronic pro-inflammatory state that impairs insulin sensitivity. Inflammasome activation can be induced by hyperglycemia, reactive oxygen species, palmitate, lipopolysaccharides, and uric acid, among other substances [24]. These findings highlight the potential molecular intervention in pathways regulating caspase-1 activation for management of chronic inflammation [29–31, 33].

Recent studies show that a protein upregulated by glucose, the thioredoxin interacting protein (TXNIP), interacts with NLRP3, leading to IL-1*β* secretion and hampering of pancreatic *β*-cell function [34, 35]. 

## 4. Inflammatory Cytokines

The origin of inflammation during obesity and the underlying molecular mechanisms that explain its occurrence are not yet fully understood, but pro-inflammatory cytokines play a central role. In obesity, there are higher circulating concentrations of inflammatory cytokines than in lean beings, and it is believed that they play a role in causing insulin resistance. The main source of pro-inflammatory cytokines in obesity is the adipose tissue; they are mainly produced by infiltrating macrophages, although adipocytes play a role. In this way, blood concentrations of these cytokines are lowered following weight loss [22, 23]. The main cytokines responsible of chronic inflammation are tumor necrosis factor-*α* (TNF*α*), interleukin-6 (IL-6), and the inflammasome-activated IL-1*β* mentioned earlier.

TNF-*α* is a pleiotropic molecule that plays a central role in inflammation, immune system development, apoptosis, and lipid metabolism, with numerous effects in adipose tissue, including lipid metabolism and insulin signaling. Circulating TNF-*α* is increased in obesity and decreased with weight loss. TNF-*α* promotes the secretion of other powerful pro-inflammatory cytokine, IL-6, and reduces anti-inflammatory cytokines like adiponectin. TNF-*α* induces adipocytes apoptosis and promotes insulin resistance by the inhibition of the insulin receptor substrate 1 signaling pathway [36, 37].

IL-6 is a cytokine that plays important roles in acute phase reactions, inflammation, hematopoiesis, bone metabolism, and cancer progression. IL-6 regulates energy homeostasis and inflammation; it is capable of suppressing lipoprotein lipase activity, and it controls appetite and energy intake at hypothalamic level [38]. IL-6 is important in the transition from acute inflammation to chronic inflammatory disease. It contributes to chronic inflammation in conditions such as obesity, insulin resistance, inflammatory bowel disease, inflammatory arthritis, and sepsis when deregulated [39].

IL-1*β* is a pyrogenic cytokine. It is mainly produced by blood monocytes in response to infection, injury, or immunologic challenge; it causes fever, hypotension, and production of additional pro-inflammatory cytokines, such as IL-6. IL-1*β* is formed from its pro-IL-1*β* inactive precursor by the inflammasome. In this way, IL-1*β* has now emerged as a prominent instigator of the pro-inflammatory response in obesity [24].

Important advances have been reached in the last decade in the understanding the role of cytokines and the inflammasome in obesity, chronic inflammation, insulin resistance, and type 2 diabetes. However, further research is required to better understand the underlying mechanisms as they are potential intervention points in the search of new therapeutically modalities for these global health problems.

## 5. Markers of Inflammation

Several chronic diseases involve an inflammatory response characterized by the increase of cytokines and serum concentrations of acute-phase reactants (markers of active inflammation) such as fibrinogen, C-reactive protein (CRP), complement, serum amyloid A, haptoglobin, sialic acid and low albumin concentrations [40]. Acute-phase reactants are synthesized in the liver, and its production is regulated by cytokines, including IL-6 and TNF-alpha [41–44]. The CRP, consideredes the classic sensitive acute-phase reactant, is a very sensitive systemic marker of inflammation, and its serum concentration increases rapidly in response to a variety of stimuli. This protein is present in low concentrations under normal conditions [45, 46].

Visceral adipose tissue may produce inflammatory mediators, which induce the production of acute-phase reactants in hepatocytes and endothelial cells [47]. In fact, because it has been shown that adipocytes express and secrete TNF-alpha, adipose body mass may be an important mediator to explain the relation between obesity and inflammation [48]. Some studies have shown that abdominal adiposity is associated with elevation of CRP levels, independent of body mass index (BMI), which is a measure of general adiposity. The proportion of people with elevated hs-CRP was significantly higher in those individuals with abdominal adiposity than control subjects, although they had a similar BMI [49]. IL-6 is a pro-inflammatory cytokine synthesized by adipose tissue, endothelial cells, macrophages, and lymphocytes. The CRP is synthesized in the liver largely in response to IL-6 stimuli [50]. Individuals with obesity are at increased risk for various chronic diseases, several of which are also characterized by elevated CRP concentrations. Because adipose tissue is a major source of pro-inflammatory cytokines such as IL-6 and TNF-alpha, both cytokines increase hepatic lipogenesis [51, 52] and trigger a systemic acute-phase response [41].

In recent years, it has been demonstrated that obesity is associated with low-grade inflammatory process characterized by the increase in circulating levels of pro-inflammatory cytokines such as IL-6, TNF-alpha, and acute-phase proteins (CRP and haptoglobin) in healthy obese subjects [53–56]. This phenomenon is also observed in obese children who have higher CRP levels than normal weight children [57]. Some studies have reported that weight loss, through diet, is associated with reduction in circulating levels of IL-6, TNF-alpha, CRP, and other markers of inflammation, independently of age, sex, and BMI [58, 59]. Similarly, weight reduction observed in subjects after gastric bypass shows decrease of CRP and IL-6 levels [60].

## 6. Metabolic Syndrome

The metabolic syndrome is characterized as the presence of three or more of the following features: obesity, hyperglycemia, hypertension, low HDL cholesterol levels, and/or hypertriglyceridemia [61–64]. Although pathogenic mechanisms are poorly understood, a central role has been attributed to the pro-inflammatory cytokines TNF-alpha [65] and IL-6 [66], since both are synthesized by adipose tissue. This syndrome has been associated with markers of inflammatory activity, such as CRP [67–75], IL-6 [76, 77], serum amyloid A [78, 79], and soluble adhesion molecules [73, 75, 80, 81].

*Risk Factors*. Low-grade chronic inflammation is associated with metabolic syndrome [82] and some features of insulin resistance [83]. Other studies have demonstrated significant correlation between CRP levels with features of the metabolic syndrome, including adiposity, hyperinsulinemia, insulin resistance, hypertriglyceridemia, and low HDL cholesterol [84, 85]. Few studies have reported the association between CRP and development of metabolic syndrome [50, 86]. In addition, it has been observed that elevated hs-CRP levels are associated with increased risk for incident cardiovascular events among individuals as having the metabolic syndrome [87]. Inflammation has been proposed as common part of different metabolic disturbances of insulin, glucose, and lipids that influence the underlying development of metabolic syndrome [50]. 

Also, it has shown that CRP adds independent prognostic information on severity of metabolic syndrome [87]. Given the evidence, it has been proposed that CRP is an additional component of metabolic syndrome [88]. In one study, it was reported that elevated levels of CRP (≥3 mg/L) may increase the risk of metabolic syndrome mediated through obesity and factors related to insulin resistance [50].

*Treatment*. Observational studies have shown that dietary patterns similar to the Mediterranean diet, rich in fruit and vegetables and high in monounsaturated fats and fiber, resulted in decrease prevalence of the metabolic syndrome [89–91]. In addition, interventional studies also demonstrated a decrease in markers of inflammation in subjects with metabolic syndrome consuming Mediterranean diet and/or national dietary guidelines [92, 93].

Studies that evaluate markers of inflammation in individuals with metabolic syndrome are scarce; however, some have shown anti-inflammatory effects of statin therapy [94, 95]. Because subjects with metabolic syndrome exhibit increased inflammation, after therapeutic lifestyle changes, statins could be a therapeutic option.

## 7. Cardiovascular Disease

In the last years, different markers of inflammation (such as CRP, IL-6, and TNF-alpha, among others) have been studied in prediction of coronary events; on this regard, CRP is the most important marker for cardiovascular disease [96].

*Risk Factors*. Circulating elevated levels of inflammatory markers, such as CRP, TNF-alpha, and IL-6, are associated with increased risk of developing cardiovascular disease [97–102]; even some acute-phase reactants may also contribute to their pathogenesis [103]. Though in mild degree, chronic elevation of CRP levels, even within normal value range, is an independent predictor of future cardiovascular events [99, 104]. Stratified ranges of high-sensitivity CRP levels of <1, 1–3, and >3 mg/L correspond to low, moderate, and high risks for future cardiovascular events. Previously, some studies have found a significative association between CRP and cardiovascular risk [105, 106]. This finding was observed for the first time over 50 years ago, where increased CRP level, after myocardial infarction, was identified as marker of poor prognostic [87]. Later, the European Concerted Action on Thrombosis and Disabilities Angina Pectoris Study Group reported that CRP concentrations were higher in the patients who had coronary events than in those without such events [107]. In addition, the Cholesterol and Recurrent Events Trial showed that elevated CRP levels are associated with major risk of coronary events after myocardial infarction [108]. A growing body of evidence has corroborated that inflammation is a strong predictor of future cardiovascular events [96–99, 104, 109–114]. 

Furthermore, hsCRP is better marker of cardiovascular disease than others acute-phase reactants, cytokines, and soluble adhesion molecules [115]. Thus, supported by a large number of observational studies and meta-analyses, CRP is considered as a mediator of cardiovascular disease [116], independently of age, smoking, cholesterol levels, blood pressure, and diabetes among others traditional risk factors evaluated in the clinical setting [117]. Thus, CRP is one of the most well-documented emerging cardiovascular disease risk factors [118, 119].

*Treatment.* Some interventional studies using Mediterranean diet and others characterized by increased intake of mustard or soybean oil, fruits, vegetables, nuts, and whole grains reduced the rate of cardiovascular disease with significant anti-inflammatory effect [120, 121]. Also, various observational and interventional studies found that intake of omega-3 and omega-6 fatty acids and alpha-linolenic acid resulted in lower risk of cardiovascular disease and lower concentrations of markers of inflammation [122–128]. Moreover, several studies have shown that statin therapy is associated with reduced inflammation and cardiovascular risk reduction [108, 129–141].

## 8. Diabetes

Several studies have shown that subclinical systemic inflammation, as measured by elevated levels of CRP and IL-6, predicts the development of diabetes [142–149]. In fact, IL-6 may interfere with insulin signalling through induction of proteins that bind to the insulin receptor [150]. On this regard, a growing body of evidence supports the hypothesis that chronic systemic inflammation contributes to decrease of insulin sensitivity at peripheral tissues [40, 45, 151, 152].

*Risk Factors*. Several studies in healthy subjects have confirmed that elevated levels of CRP and cytokines IL-6 and TNF-alpha are associated with insulin resistance [84, 85, 153–155]. In addition, it has been shown that in the individuals with impaired glucose tolerance [156, 157], the low-grade chronic inflammation is related to glucose metabolic disturbances.

It has been reported that TNF-alpha is overexpressed in the adipose and muscle tissues of obese and insulin-resistant nondiabetic subjects, overexpression that is positively correlated with insulin resistance [48, 158–160]. Interestingly, circulating TNF-alpha levels are higher in type 2 diabetes [161–163] as compared with IFG/IGT [156]. In addition, several cross-sectional studies have shown an increase of CRP levels in patients with diabetes [142, 143, 164] and the increase of CRP, IL-6, and TNF-alpha in subjects with IGT [40, 165].

Moreover, in obesity there are elevated levels of several kinases such as protein kinase C isoforms, I Kappa B Kinase-*β*, and c-jun-terminal kinase, and these kinases have been implicated in alteration of insulin signaling by promoting serine phosphorylation of insulin receptor substrate which is associated with suppression of tyrosine phosphorylation of this substrate [166]. Also, various studies have demonstrated that nutrient excess and obesity are associated with elevated levels of free fatty acids, which can induce both insulin resistance in peripheral tissues and activation of innate immunity [28, 167–172].

Furthermore, it is difficult to set cut-point values to predict risk of development disease because intermediate values of CRP are at moderate risk for metabolic disturbances. However, it has been reported that patients with diabetes and CRP values >3 mg/L have 51% higher risk of all-cause mortality and 44% higher risk of cardiovascular mortality than subjects with diabetes and CRP <3 mg/L of similar age and sex, independently of classical risk factors such as lipids, blood pressure, and glycemia [173].

*Treatment*. In clinical field, there are different therapeutic options, such as genetic, biochemical, and pharmacological targeting of inflammatory signalling pathways improving insulin action, a central problem in the pathophysiology of type 2 diabetes [174]. Existing evidence about inhibiting specific inflammatory kinases pathway improves insulin action in animal models [175, 176]. Pharmacological therapeutics using thiazolidinediones exhibited anti-inflammatory effects inhibiting both adipocyte and macrophage function in obesity and type 2 diabetes [177]. Various clinical studies, using anti-inflammatory drugs to treat type 2 diabetes and even prediabetes, showed improvements in beta-cell function and insulin sensitivity, reducing glucose levels [34, 178–182]. In addition, others studies in patients with type 2 diabetes taking statins have demonstrated a beneficial and additive effect on markers of inflammation [183–186], which could be an alternative therapeutic for this disease; however, the clinical practice recommendations should be considered about the appropriate use of statin therapy because basic studies have documented controversial results regarding the beneficial and adverse effects on insulin secretion and sensitivity [187].

## 9. Conclusion

The origin of inflammation during obesity and the underlying molecular mechanisms that explain its occurrence are not still fully understood, but pro-inflammatory cytokines play a central role. In obesity, there are higher circulating concentrations of inflammatory cytokines than in lean beings, and it is believed that they play a role in causing insulin resistance. The main source of proinflammatory cytokines in obesity is the adipose tissue; they are mainly produced by infiltrating macrophages, although adipocytes play a role. Obesity is a consequence of many risk factors, as increased energy consumption and reduced physical exercise. Many problems ecist in patients with obesity, as cardiovascular disease, diabetes, metabolic syndrome, and NAFLD, among others, predicting the risk of future cardiovascular events and mortality. Different mechanisms, including antioxidant, anti-inflammatory, fiber diet, and antiestrogenic processes, have been proposed to explain the protective nature of certain dietary components, particularly, components of Mediterranean diet which could be an important therapeutic lifestyle change which allows to avoid the development of metabolic diseases.
